# Downstream Approach Routes for the Purification and Recovery of Lactobionic Acid

**DOI:** 10.3390/foods11040583

**Published:** 2022-02-17

**Authors:** Inga Sarenkova, Sara Sáez-Orviz, Manuel Rendueles, Inga Ciprovica, Jelena Zagorska, Mario Díaz

**Affiliations:** 1 Faculty of Food Technology, Latvia University of Life Sciences and Technologies, 22a Rigas Street, LV3004 Jelgava, Latvia; inga.ciprovica@llu.lv (I.C.); jelena.zagorska@llu.lv (J.Z.); 2 Department of Chemical Engineering and Environmental Technology, Faculty of Chemistry, University of Oviedo, Av. Julian Clavería 8, 33006 Oviedo, Spain; saraorviz@hotmail.es (S.S.-O.); mrenduel@uniovi.es (M.R.); mariodiaz@uniovi.es (M.D.)

**Keywords:** lactobionic acid, whey, freeze drying, downstream approaches

## Abstract

The successful development of a lactobionic acid (LBA) bioconversion process on an industrial scale demands the selection of appropriate downstream methodological approaches to achieve product purification once the bioconversion of LBA is completed. These approaches depend on the nature of the substrate available for LBA production, and their necessary implementation could constitute a drawback when compared to the lesser effort required in downstream approaches in the production of LBA obtained by chemical synthesis from refined lactose. Thus, the aim of this research is to separate LBA from an acid whey substrate after bioconversion with *Pseudomonas taetrolens*. Freeze drying, crystallization, adsorption with activated carbon, microfiltration, centrifugation, and precipitation with 96% (*v*/*v*) ethanol were carried out to separate and purify LBA. The closest product to commercial LBA was obtained using precipitation with ethanol, obtaining a white powder with 95 ± 2% LBA concentration. The procedure described in this paper could help to produce LBA on an industrial scale via microbial bioconversion from acid whey, developing a promising biotechnological approach for lactose conversion.

## 1. Introduction

Lactobionic acid (LBA), a versatile PHA (polyhydroxy acid) containing two hydroxyl groups per molecule, has as received commercial recognition as a compound with numerous auspicious applications in the food, cosmetics, chemical and pharmaceutical industries, as well as in medicine [1,2]. Because of its wide application, the production value of LBA is increasing worldwide [3].

Presently, LBA is mainly obtained from lactose by chemical, electrochemical, heterogenic or biocatalytic synthesis. These processes are labor intensive and expensive [4]. LBA production by microbial enzymatic routes offers extremely promising systems able to take advantage of the costs and benefits of using by-products, such as whey. However, as most biological systems, these methods still need to be improved so that they overcome the connate barriers to the process and achieve efficiency [5,6]. The biotechnological approach to LBA production from whey not only reduces the production costs, but also plays a significant role in the bio-remediation of waste [7]. The research findings indicate that the microbial bioconversion of lactose into LBA with *Pseudomonas taetrolens* is successful when using whey as a substrate [5].

However, when the upstream part of the production process is completed effectively by means of bioconversion, the overall capability of the process can become limited by the downstream part and the approach employed in this paper, in the purification stages, can have an enormous impact on the final price of the product [3].

Methods of purification and separation have evolved over the years, and procedures, such as precipitation with ethanol [8,9], evaporation, crystallization, and electrodialysis, have been replaced by ion-exchange chromatography, obtaining 100% yield of LBA and fast identification of the constituents by high performance liquid chromatography (HPLC) [3,10]. Crystallization after precipitation with ethanol has also been proposed as a purification and recovery step after the LBA production part [11].

It is important to separate the biomass immediately after the fermentation process, and the physical removal of microorganisms by microfiltration can be employed. The pressure of microfiltration and velocity of the fluid are key factors affecting performance in microfiltration and that enable a cleaner recovery product to be obtained [12]. Product drying after microfiltration has also been proposed for the production of crystalline and syrup form LBA [13]. Gutiérrez et al. [14] successfully tested a strongly acidic commercial cation exchange resin (AmberliteTM FPC23 H type) to produce LBA from sodium lactobionate, separating almost all of the sodium. Chromatography methods enable the fractionation of carbohydrate solutions, producing separate products with a purity of ≥99% [3].

Different downstream routes for LBA dissociation from dairy waste are the focus of the current study.

## 2. Materials and Methods

### 2.1. LBA Substrate Preparation

Acid whey (from artisan cheesemaker Ca Sanchu, Asturias, Spain) with the following parameters—pH 4.57 ± 0.06, lactose 4.20 ± 0.14%, proteins 0.14 ± 0.04% and fats 0.01 ± 0.01%—was microfiltered and clarified. *Pseudomonas taetrolens* LMG 2336 was maintained frozen at −20 °C in 40% (*v/v*) glycerol (Belgian Coordinated Collection of Microorganisms, Ghent, Belgium). The *Pseudomonas taetrolens* strain was cultured on Nutrient Broth (NB) with 1 g L^−1^ meat extract, 2 g L^−1^ yeast extract, 5 g L^−1^ NaCl, 5 g L^−1^ peptone (all from Sigma-Aldrich, Steinheim, Germany) and 20 g L^−1^ agar (VWR Chemicals, Radnor, PA, USA). The agar plates were incubated at 30 °C for 48 h and used directly for the preparation of inoculum. Erlenmeyer shaker flasks containing 100 mL of NB were inoculated with a loopful of 10 µL of *Pseudomonas* *taetrolens* from the NB agar plates. These samples were incubated for 10 h at 30 °C in an orbital shaker with an agitation rate of 350 rpm (New Brunswick Sci., Edison, NJ, USA). The biomass was separated by centrifugation at a rate of 10,000 rpm for 10 min and then used as a bulk starter. Acid whey was inoculated with 30% *v/v Pseudomonas taetrolens* and bioconverted in a bioreactor (BioFlo 110, New Brunswick Scientific, Edison, NJ, USA) at a temperature of 30 °C for 72 h with 1.5 Lpm aeration rate, 350 rpm agitation and maintaining the pH at 6.5 with 6 M NaOH during bioconversion [15].

After the bioconversion process, the substrate did not contain lactose (based on HPLC analysis). The final sample from the bioconversion was further subjected to purification processes.

### 2.2. Downstream Approaches

The sample was further divided into four individual samples. Downstream approaches differed for each sample (Table 1).

- Centrifugation—After lactose bioconversion in the bioreactor, the sample was centrifuged for 10 min at 15,000 rpm to remove biomass and other water-insoluble substances.
- Activated carbon—After bioconversion, samples were treated using activated carbon (Norit GAC 1240, Amersfoort, The Netherlands). After treatment, the samples were centrifuged for 10 min at 15,000 rpm to remove the activated carbon and other water-insoluble substances.
- Microfiltration—The samples were treated using microfiltration to remove the biomass with a tangential microfiltration device with a polyvinylidene fluoride (PVDF) membrane cassette (0.5 m^2^ membrane area, Pellicon 2 cassette) with a 0.22 μm pore size (Millipore, Billerica, MA, USA), a transmembrane pressure of 1.4 ± 0.1 bar, and without recirculation.
- Evaporation—Samples were concentrated until reaching a concentration of solids of ~40% in a “Heidolph Laborota 4000 efficient” (Heidolph Instruments GmbH & Co KG, Schwabach, Germany) evaporator. The process was carried out at 50 °C, with flask stirring speed of 150 rpm, pressure 40 to 85 mbar. The concentration of the sample was detected with a refractometer (Kruss, Hamburg, Germany).
- Precipitation with ethanol-concentrated samples (~40% total solids) were rinsed with 96% (*v/v*) ethanol. After rinsing, the samples were centrifuged at 10,000 rpm for 10 min to precipitate the LBA. The remaining solvent was separated by pouring off, and then the samples were crystallized [9,16].
- Crystallization—samples were crystallized in silicone cups by drying them at a temperature of +40 °C for 24–72 h in an oven.
- Freeze drying—concentrated samples (~40% total solids) with a volume of 100–200 mL were stored in a freezer at −18 °C for >24 h. Frozen samples were freeze dried to a powdered physical state in vacuum lyophilization equipment (Telstar cryodos—80, Model 2007, Terrassa, Spain) at a temperature of −65 °C.

### 2.3. Analytical Methods

The obtained LBA products were compared with commercial chemically synthesized LBA purchased from “Sigma-Aldrich”, (Saint Louis, MO, USA; the company indicates that this product contains >97% LBA) and henceforward referred to as “commercial LBA”, and with commercial lactose purchased from “Pa Panreac” (Barcelona, Spain, containing > 99% lactose), which is in this paper referred to as “commercial lactose”.

- The recovery of LBA was calculated as percentage by dividing the amount of LBA collected after downstream approaches by the amount of LBA in fermented substrate before downstream approaches, and then multiplying this number by 100 [17].
  (1) $$\mathrm{Recovery},\;\%=\frac{{\mathrm{LBA}}_{After}\;}{{\mathrm{LBA}}_{Before}}\times 100$$
  where:
  LBA*_Before_*—amount of LBA in fermented substrate before downstream approaches, g L^−1^;
  LBA*_After_*—amount of LBA collected after downstream approaches, g L^−1^.
- pH was determined by dissolving 1 g of the analyzed product in 10 mL of deionized water (10% solution) and measured with an InLab^®^ Expert Pro-ISM pH electrode (Mettler Toledo, Greifensee, Switzerland). Before analyses, the pH electrode was calibrated using buffers with known pH (4.01, 7.00, and 10.00, WTW^TM^, Wolverhampton, England), by matching pH meter to the current characteristics of pH sensor with reliable accuracy to the second decimal of pH.
- Color analysis was conducted by dissolving 1 g of analyzed product in 10 mL of deionized water (10% solution). Color analysis was performed using L*a*b* coordinates defined by the International Commission on Illumination with a “Lovibond^®^ LC100” (Tintometer^®^ group, Lovibond house, Amesbury, UK) color analyzer. A negative a* value indicates the intensity of the green color, a positive value of a* indicates the intensity of the red color, a negative value of b* indicates the intensity of the blue color, a positive value of b* indicates the intensity of the yellow color, and L* is an indicator of white and black or light and dark intensity. The total color intensity difference$\;—\Delta E_{ab}$ was calculated by comparing the color of the obtained product with the color of commercial LBA. The given formula calculates the difference between the two colors to identify inconsistencies [18].
  (2) $$\Delta E_{ab}=\sqrt{{\left(L_{2}-L_{1}\right)}^{2}+{\left(a_{2}-a_{1}\right)}^{2}+{\left(b_{2}-b_{1}\right)}^{2}}$$
  where:
  *L*_2_*, a*_2_*, b*_2_—color of the evaluated product;
  *L*_1_*, a*_1_*, b*_1_—color of commercial LBA.
  The color analyzer was calibrated for color intensity analyses. The samples were poured into equal volumes in transparent containers and placed at the analyzer sensor. The lid was closed above the containers so that ambient light had no effect on the color of the samples. Results are expressed as the mean  ±  standard deviation of seven experiments.
- Solubility in water was determined by adding 0.1 g of obtained product to 10 mL of deionized water at a temperature of +20 °C and stirring the product at 150 rpm until completely dissolved. Obtained product solubility was determined by time measured in seconds [19].
- LBA and lactose concentration were detected by high-performance liquid chromatography (HPLC, Agilent 1200, Agilent Technologies Inc., Santa Clara, CA, USA), employing a column (Coregel-ION 300, Teknocroma, Barcelona, Spain) coupled to a refractive index detector at +40 °C. Sulphuric acid (0.450 mM, pH 3.1) was used as the mobile phase, with a column temperature of +75 °C and a flow rate of 0.3 mL min^−1^. All obtained samples before HPLC analyses were dissolved in deionized water at +20 °C and centrifuged for 10 min at 15,000 rpm to remove cell debris and other water-insoluble substances [15].
- Protein determination was measured by the Kjeldahl method (ISO 8968-1:2014) using KjeltecTM 2200 (FOSS, Sweden).
- LBA concentration in obtained LBA products (Sample A, B, C or D) was calculated as percentage by dividing the mass of the pure LBA (determined by HPLC) by the total mass of the sample (obtained LBA product: sample A, B, C or D), and then multiplying this number by 100 [20]:
  (3) $$\mathrm{LBA}\;\mathrm{concentration},\;\%=\frac{{\mathrm{LBA}}_{HPLC}\;}{{\mathrm{LBA}}_{product}\;}\times 100$$
  where:
  LBA*_product_*—amount of LBA product, g;
  LBA*_HPLC_*—amount of LBA by HPLC analyses in LBA product, g.
- Product visual assessment was carried out immediately after the performance of downstream approaches and after storage of the unpackaged product at room temperature for 24 h.

### 2.4. Stastistical Analysis

Results are expressed as the mean  ±  standard deviation of three experiments unless specified otherwise. Data acquisition and analysis were performed with the variance analysis method ANOVA, and a t-test was performed. A *p* value lower than 0.05 (*p* <  0.05) was considered significantly different. Data acquisition and analysis were performed with ChemStation software (Agilent) and Microsoft Office Excel v16.0.

## 3. Results and Discussion

**Figure 1:**
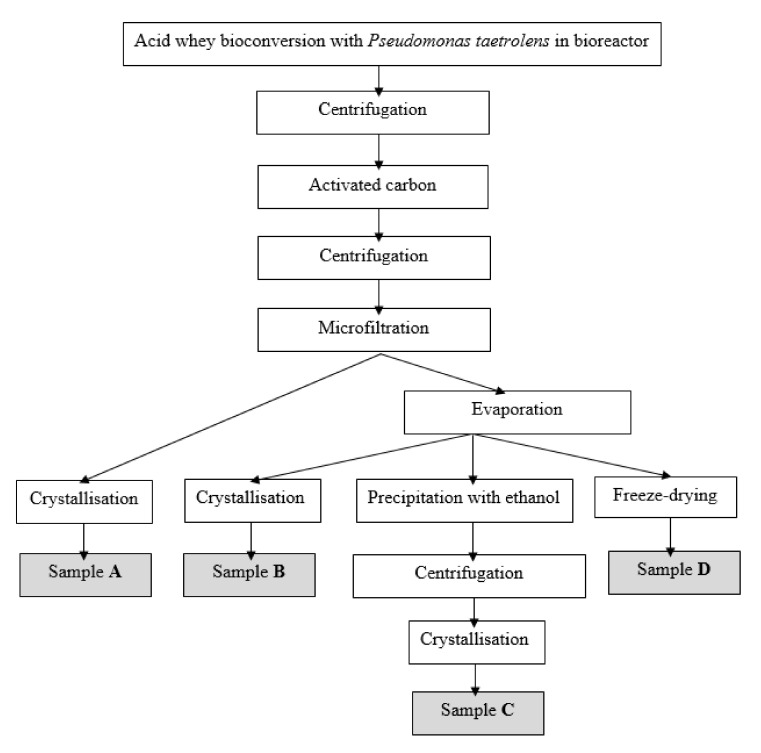
Flow diagram of downstream approach routes.

The LBA samples were subjected to different downstream steps (Figure 1), and therefore the obtained LBA products differ visually. The flow of the proposed downstream steps is summarized in Figure 1; it is important to purify the substrate by removing soluble impurities with activated carbon and cell debris and other water-insoluble substances with centrifugation and microfiltration steps, before performing crystallization, precipitation with ethanol and freeze drying.

Centrifugation separates the components of the substrate according to the Stokes equation, which states that centrifugation depends on particle density, diameter and size. Thus, this downstream step after the bioconversion process is important to achieve the separation of the main amount of *Pseudomonas taetrolens* biomass from substrate containing LBA [21]. Complete biomass separation is performed with microfiltration. Additionally, Sáez-Orviz et al. [22] have proved that microfiltration eliminates endotoxins from sweet whey substrate after fermentation with *Pseudomonas taetrolens*, which means that the obtained LBA product is safe for use in the food industry.

### 3.1. Decolouring Efficiency of Activated Carbon

**Figure 2:**
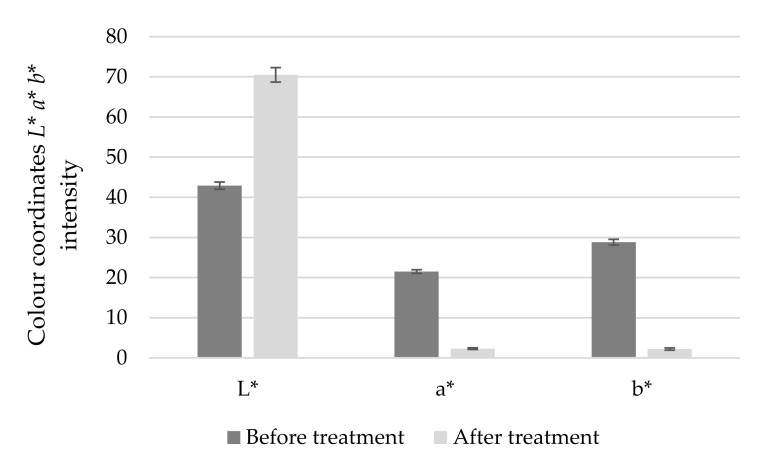
Changes in color intensity in L* coordination of white-black color; a* coordination of red-green color; b* coordination of yellow-blue color.

After bioconversion, the substrate was treated with activated carbon, in order to separate the unwanted color pigments. As a result, the color of the substrate changes from yellow to transparent. The sample became lighter and lost the noticeable intensity of red and yellow color (Figure 2), as in a study of Sulaymon and Abood [23], who showed that yellow dye was removed as an adsorbate onto activated carbon from wastewaters. Activated carbon has also been used for the decolorization of sugar cane juice and can be a good decoloring agent for citric acid fermentation [24].

The adsorption takes place on the inner walls of the pores in the particles. Activated carbon efficiently adsorbs high molecular weight and low solubility compounds. Adsorption by activated carbon is most effective in removing organic matter, the degree of effectivity depending on the composition of the compounds in question [25,26]. The observed differences are significant (*p* < 0.05) before and after treatment in all color L*a*b* system coordinate intensities. Adsorption with activated carbon helps to obtain a cleaner product and divest it of inappropriate color, thus this step is important to the recovery of LBA after bioconversion.

### 3.2. Assessments of LBA Samples

**Figure 3:**
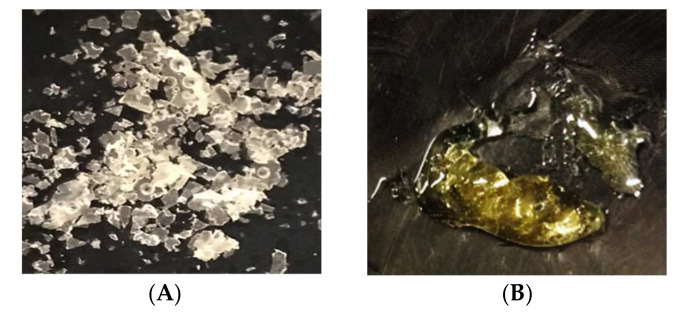

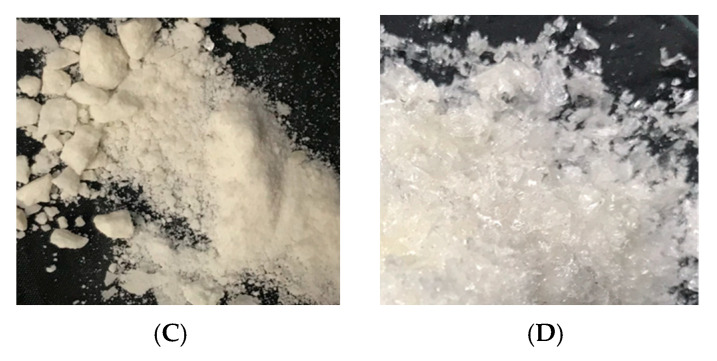
Lactobionic acid product visual assessment immediately after the performance of downstream approaches. (**A**) Light yellow shades, free-flowing crystalline product; (**B**) Yellow shade syrup (visually reminiscent of honey); (**C**) Light, white colored fluid powder-like product; (**D**) Light, white colored fluid crystalline product.

The appearance of LBA products is shown in Figure 3. All LBA samples took the form of crystals except for sample B, which had a syrup-like consistency. This sample was obtained by evaporation followed by crystallization in a drying oven; the resulting material was dense and the effective surface area available to vaporize water in the drying oven was small compared to sample A, which did not undergo the evaporation step and was therefore crystallized from an initial liquid state. Wilkinson et al. [27] have also established that products in a gel state took longer to dry than those in liquid state.

In the visual assessment the closest to commercial LBA is sample D, which was obtained by the freeze-drying process.

In all the obtained LBA samples no protein was detected by the Kjeldahl method, and they were shown to be lactose-free by HPLC. The composition of the impurities in the LBA products could be minerals, vitamins, cell debris, water, and other compounds [15], but analyses to determine these compounds were not carried out.

Commercial LBA also showed structural changes after 24 h storage of unpacked products at room temperature (Table 2); the LBA crystals were hygroscopic and sticking of the crystals was observed. Similar trends were seen with samples A and D, while samples B and C retained their appearance during 24 h storage.

Delagustin et al. [19] stated that the physical characteristics of LBA had changed from powder to gel after 6 months of storage; an increase of approximately 12% was observed in the moisture content. The water retention is attributed to the hygroscopic nature of LBA [28]. Shendurse and Khedkar [29] have also emphasized that LBA is hygroscopic and forms a gel containing around 14% water. X-ray diffractometry was performed in the research of Bisinella et al. [30], who found that stored unpacked LBA presented a decrease in crystallinity, which was attributed to high LBA hygroscopicity (with low angles at 2θ: 18.96° and 19.34°).

Cardoso et al. [2] have reported that the moisture content of an LBA product should be below 4.68% in order to qualify as a powder; higher moisture content can give the appearance of a defective product (gel structure) and shorten the shelf life.

The LBA amount of all samples before downstream approaches was 39.5 ± 1 g L^−1^, obtained after a fermentation process carried out in a bioreactor (more details of the fermentation process are specified in Sarenkova et al. [15]). After the downstream approaches, sample C had a lower LBA recovery yield than other samples (*p* < 0.05), while samples A, B and D presented the same yield. The maximum of 100% recovery was not attained in this study, and it would appear that microfiltration, activated carbon and centrifugation caused losses during the recovery process, as did precipitation with ethanol (Table 2). After fermentation, Murakami et al. [9] also added ethanol to the fermented substrate, resulting in 98% LBA recovery. Jones and Ho [31] proposed an optimized method for LBA recovery involving a crystallization step and using a precipitation method and a final ion-exchange process. In the precipitation stage, the precipitate was passed via one or more ion-exchange resins before a final freeze-drying process, resulting in a 79% recovery of LBA. While Pedruzzi et al. [32], Splechtna et al. [33] and Borges de Silva et al. [17] reached 100% recovery yield of LBA by using ion exchange chromatography or simulated moving bed technology, Peretti et al. [34] reached only 38.7% recovery yield of LBA by using electrodialysis.

### 3.3. Results of Colour Analysis

**Figure 4:**
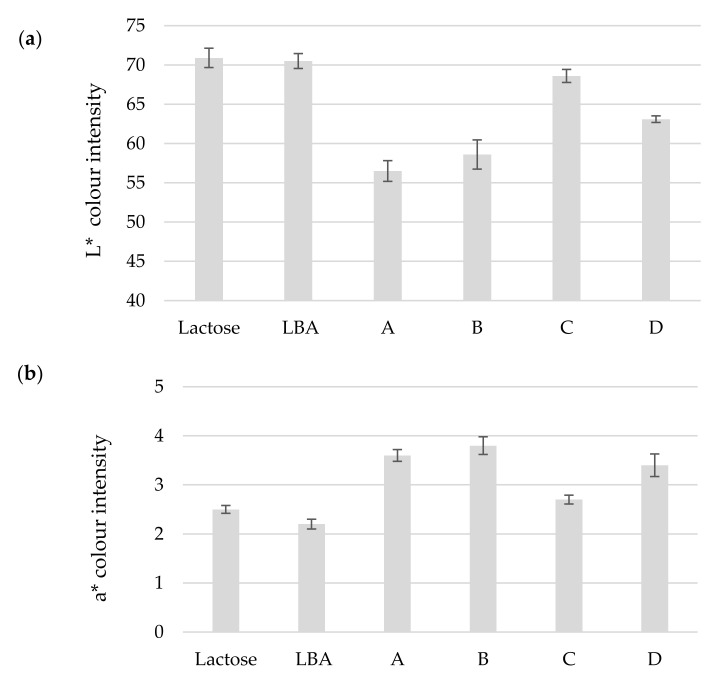

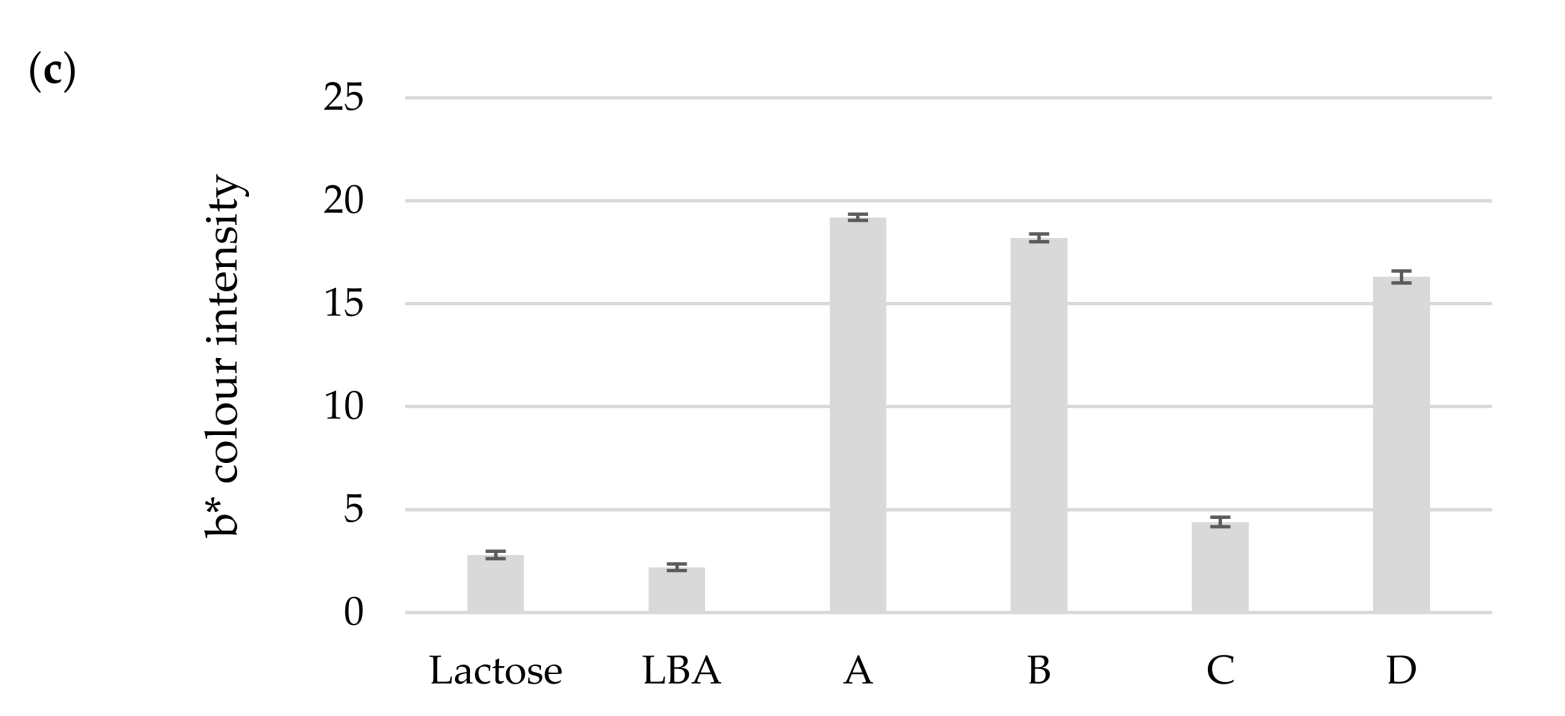
Results of color analysis of “Pa Panreac” lactose, “Sigma-Aldrich” lactobionic acid (LBA) and lactobionic acid samples. (**a**) L* coordination of white-black color; (**b**) a* coordination of red-green color; (**c**) b* coordination of yellow-blue color.

LBA is obtained by oxidizing lactose [5] and so the color of the experimental LBA samples was compared with that of commercial lactose and LBA. The appearance of the product gives a hint as to the presence of impurities which influence its color and Figure 4 shows a comparison of color intensities between samples.

In Figure 4a, it can be observed that samples A, B and D are darker than commercial lactose and LBA (*p* < 0.05), but sample C has the same white intensity as commercial LBA and lactose (*p* > 0.05). Furthermore, in Figure 4b,c, it is clear that all obtained LBA samples have a higher red and yellow color intensity compared to the commercial LBA and lactose (*p* < 0.05). Color analysis shows that the obtained samples contain impurities, which give them this hue. In the color wavelengths tested, the closest to commercial LBA is sample C compared to other LBA samples; the downstream approach with precipitation in ethanol seems to help to divest the recovered LBA of its uncharacteristic color.

No reports were found regarding the color analysis of LBA, although Delagustin et al. [19] have mentioned that LBA obtained via bioconversion in a bioreactor and after the purification and recovery steps is a white powder, defining it as transparent, but during the stability test the color could change from transparent to light brown [19]. Additionally, Illanes et al. [10] classified LBA as a bionic acid, which is a white-colored powder in crystallized form.

### 3.4. LBA pH, Solubility, Concentration and Difference in Colour Intensity

LBA concentration, color intensity, pH and solubility in water were determined (Table 3) to compare the compliance and purity of samples with commercial LBA. LBA concentration detected by HPLC is very close to the commercial LBA product. LBA concentration in sample C corresponds to commercial LBA (97%). Sample B contains less LBA than other samples; it could be due to the syrup-like consistency and the result for LBA concentration was significantly different (*p* < 0.05) from other analyzed samples.

Sample C was made by precipitation with 96% (*v/v*) ethanol and drying at a temperature of +40 °C; the same method was applied in the research of Delagustin et al. [19], who added 70% (*v/v*) ethanol, followed by drying at a temperature of +25 °C, obtaining a sample with 93.71% LBA. A total of 95 ± 2% LBA was obtained by precipitation with ethanol; it seems that centrifugation, microfiltration and treatment with activated carbon before precipitation with ethanol increased recovery and improved the purification process of LBA.

In terms of pH, sample C is closest to commercial LBA. All obtained LBA samples were significantly different in pH from commercial LBA (*p* < 0.05). Delagustin et al. [19] reported that the pH values of LBA (obtained after precipitation with ethanol) and commercial LBA remained at around 2.5. Similar results were also described by Carra [35], where a pH of 2.96 was determined for a freeze-dried LBA product and 3.04 for commercial LBA (Sigma-Aldrich). Cardoso et al. [2] have reported that LBA has pH 2.37 (10% solution). Sample B has the highest pH among all samples (*p* < 0.05); the main reason could be the moisture content in the sample.

Sample A and D have the same solubility as commercial LBA (*p* > 0.05), while sample B and C took 3–4 times longer to completely dissolve (*p* < 0.05). Sample B has a syrup-like consistency, thus the surface where water can act to dissolve it is more reduced, while in sample C, the obtained product has a denser aspect than the others, suggesting that the explanation is the same as in sample B—the product surface area is smaller than in sample A and D, where the product is fully accessible to water exposure and dissolves faster. Delagustin et al. [19] have mentioned that in their research that 1 g LBA was solubilized in 1–10 mL water, being classified as completely soluble, and according to the British Pharmacopeia Commission (2009), LBA is classified as freely soluble in water. Cardoso et al. [2] have reported that LBA has a water solubility of 10 g 100 mL^−1^. However, LBA is poorly soluble in organic solvents, such as methanol, ethanol and glacial acetic acid [2,14]. All obtained samples were easily dissolved in deionized water.

$\Delta E_{ab}$ calculation of the total color intensity difference shows that sample C is closest to the chemically synthesized LBA commercial sample. The same was observed in Figure 4 where, in each of the color intensities, the closest to commercial LBA was sample C, which was obtained by precipitation with ethanol; it seems that the sample interacts with ethanol, which dissolves some inappropriate compounds (such as vitamins and minerals), thus giving an LBA product with higher purity. This is also shown by the LBA concentration in the product, where sample C and D showed a higher yield of LBA than other samples.

## 4. Conclusions

The most appropriate method for LBA purification and recovery from a fermented substrate is precipitation with ethanol, which showed comparable physical and chemical properties to commercial LBA. Activated carbon adsorption provided a significant level of purification of the substrate from undesirable color pigments.

Crystallization and freeze-drying approaches changed the format of the substrate from liquid to syrup or crystals, while precipitation with ethanol helped to obtain a cleaner product, attaining a lactobionic acid concentration (95 ± 2%) close to that of commercial lactobionic acid (>97%). Additionally, the total color intensity difference and pH of the lactobionic acid sample treated by precipitation with ethanol showed the values closest to commercial lactobionic acid, but this sample had the smallest recovery yield.

This research opens new perspectives for the industrial production of LBA via the bioconversion of acid whey. The samples should be analyzed further for the presence of other trace elements if they are to compete with the purity and safety criteria for chemically synthesized LBA and to be safe for application in the food industry. The downstream approaches should be studied in depth in terms of cost-effectiveness on an industrial scale.

## Figures and Tables

**Table 1:** Downstream approaches used in the study.

Sample Code	Centrifugation	Activated Carbon	Microfiltration	Evaporation	Precipitation with Ethanol	Crystallization	Freeze Drying
A	✓	✓	✓	-	-	✓	-
B	✓	✓	✓	✓	-	✓	-
C	✓	✓	✓	✓	✓	✓	-
D	✓	✓	✓	✓	-	-	✓

(✓)—Indicates that indicated downstream approach was used; (-)—Indicates that indicated downstream approach was not used.

**Table 2:** Sample characteristics.

Sample Code	LBA*_Before_,* g L^−1^	Recovery of Lactobionic Acid, %	Product Description: After 24 h of Unpacked Product Storage
A	39.95 ± 1	89 ± 3 ^a^	Very hygroscopic, loses its fluid consistency, absorbs moisture quickly and a syrup-like product is formed.
B	39.95 ± 1	87 ± 2 ^a^	When stored unpacked at room temperature, it retains its consistency.
C	39.95 ± 1	82 ± 2 ^b^	When stored unpacked at room temperature, it retains its consistency.
D	39.95 ± 1	87 ± 2 ^a^	Hygroscopic, absorbs moisture quickly and loses the consistency of the fluid product. The crystals stick together and form a solid mass.

Different superscripts within a column (a, b) are significantly different (*p* < 0.05). LBA*_Before_*—amount of lactobionic acid in fermented substrate before downstream approaches, g L^−1^.

**Table 3:** LBA samples pH, solubility, concentration and total color intensity difference.

Sample Code	Sample pH	Sample Solubility, Time, s	LBA Concentration, %	Total Color Intensity Difference $\Delta E_{ab}$
A	3.48 ± 0.08 ^b^	31 ± 3 ^c^	90 ± 1 ^b^	22.60 ± 0.94 ^a^
B	4.27 ± 0.12 ^a^	118 ± 2 ^a^	85 ± 3 ^c^	20.00 ± 1.42 ^b^
C	3.05 ± 0.09 ^c^	109 ± 3 ^b^	95 ± 2 ^a^	2.95 ± 0.23 ^d^
D	3.22 ± 0.08 ^c^	23 ± 4 ^d^	94 ± 2 ^a,b^	15.97 ± 1.22 ^c^
Commercial LBA	2.32 ± 0.01 ^d^	31 ± 4 ^c,d^	-	-

Different superscripts within a column (a, b, c, d) are significantly different (*p* < 0.05).

## Data Availability

The datasets generated during and/or analyzed during the current study are available from the corresponding author upon reasonable request.
